# Patient-Reported Outcome Measures for Patients with Upper Extremity Arthritis: Overview of Systematic Reviews

**DOI:** 10.1177/11795441231213887

**Published:** 2023-12-06

**Authors:** Armaghan Dabbagh, Christina Ziebart, Rochelle Furtado, Eleni C Boutsikari, Joy C MacDermid

**Affiliations:** 1 Department of Health and Rehabilitation Sciences, Faculty of Health Sciences, Elborn College, Western University, London, ON, Canada; 2 Collaborative Program in Musculoskeletal Health Research, Bone and Joint Institute, Western University, London, ON, Canada; 3 Department of Hygiene, Epidemiology and Medical Statistics, National and Kapodistrian University of Athens, Athens, Greece; 4 Department of Physical Therapy, Faculty of Health Sciences, Elborn College, Western University, London, ON, Canada; 5 Roth McFarlane Hand and Upper Limb Centre, St. Joseph’s Hospital, London, ON, Canada

**Keywords:** Arthritis, osteoarthritis, rheumatoid arthritis, reliability, validity, responsiveness, systematic review

## Abstract

**Background:**

Arthritis leads to disabilities impacting patients’ physical and mental health.

**Objective:**

To synthesize the evidence on measurement properties of the patient-reported outcome measures (PROMs) for people with upper extremity arthritis.

**Design:**

Overview of systematic reviews (SRs).

**Methods:**

We performed an electronic search of 6 databases to retrieve SRs looking at any measurement property related to PROMs for people with upper extremity arthritis. Two authors rated the risk of bias (ROB) of the included SRs using AMSTAR. We extracted data on measurement properties from each SR.

**Results:**

From 6 included SRs, 6 PROMs (Arthritis Impact Measurement Scale [AIMS], AIMS-2, AIMS-Short Form, Cedars-Sinai Health-Related Quality of Life for Rheumatoid Arthritis (CSHQ-RA), Revised CSHQ-RA, and Influence of Rheumatic Disease on General Health and Lifestyle) were evaluated in 2 or more SRs. The ROB of the included SRs ranged from moderate to high. Low- to moderate-quality evidence was found of good construct and criterion validity, acceptable content validity, and good responsiveness of the AIMS. We found low- to moderate-quality evidence of good internal consistency, test-retest reliability, and construct validity of the CSHQ-RA.

**Conclusion:**

We found a moderate to high ROB in the included SRs on the upper extremity PROMs for patients with arthritis. More evidence was specific to upper extremity arthritis in measures not in common use versus well-validated measures used in upper extremity conditions and recommended in current core sets. These factors suggest an urgent need for additional research to improve the scope and quality of evidence before recommendations can be made specific to patients with arthritis.

**Registration Number:**

on PROSPERO CRD 42019137491

## Introduction

Arthritis limits patients’ everyday life with pain, functional disability, fatigue, and mental well-being,^1^ leading to physical and mental^2,3^ burdens. Recent research is evaluating major comorbidities^4,5^ and side-effects that secondarily arise due to arthritis or antirheumatic immunosuppressive treatment,^6-8^ describing further the overall health and socioeconomic imprint of the disease.^7^

Systematic reviews (SRs) consolidate the complexity of the diagnosis of arthritic disorders, a procedure that consists of algorithms, medical imaging, physicians’ assessment, and patient-reported outcome measures (PROMs).^8^ Patient-reported outcome measures of arthritic disorders demonstrate variations in terms of psychometric properties.^9,10^ Although certain methodological limitations arise from their use, they have proved useful prognostic tools^3^ widely developed and validated to be used in clinical practice^2,11^ to support the optimal treatment of arthritis.^12^

A wide range of clinical dimensions is assessed by PROMs, namely pain, physical functions, quality of life, mental functions, social functions, and general health.^9,10,13,14^ However, findings from single psychometric studies that examine specific scales and populations cannot be easily applied to decision-making since individual papers provide a limited evaluation of specific measurement properties on a limited number of PROMs. Previous overviews of SRs have addressed other more generic PROMs of the upper limb, such as the Disability of the Arm, Shoulder, and Hand, the Michigan Hand Questionnaire, and the Patient-Rated Wrist Evaluation.^15^ There is a lack of synthesized evidence in the form of an overview of PROMs specific to upper extremity arthritis. The purpose of this study was to address the literature gap regarding the synthesis of the psychometric properties of arthritis-specific PROMs used in assessing outcomes in patients with upper extremity arthritis.

## Materials and Methods

### Study design

This is an overview of SRs, namely a review containing SRs that assess the psychometric properties of arthritis-specific PROMs used to evaluate outcomes in patients with upper extremity arthritis. An overview is a procedure to synthesize the findings from multiple SRs that fit prespecified eligibility criteria.^16^ This study has been registered on PROSPERO, with the registration number CRD 42019137491.

### Search method

We performed an electronic search of 6 databases (MEDLINE, ILC, Embase, the Cochrane Central Register of Controlled Trials, CINAHL, and LILACS) in September 2019 for relevant literature for inclusion. We searched from the respective inception dates of the database. The search strategy was designed to locate SRs that addressed at least one psychometric property of patient outcomes used for patients with arthritis. We used keywords related to 3 concepts: (1) psychometric properties (reliability, validity, responsiveness, etc.), (2) patient-reported tools (questionnaire, patient-rated, patient-reported, pain measurement, outcome measurement, etc.), and (3) upper extremity arthritic conditions (osteoarthritis, rheumatoid arthritis [RA], thumb arthritis, etc.). We then used the “AND” function to merge the concepts. We did not impose any other restrictions regarding publication date, language, sex, gender, and so on. We updated the search in September 2022, and no new studies were added.

### Study selection

We entered the retrieved articles into EndNote X9 (Clarivate Analytics, Boston, MA, USA) and 2 independent authors (RF and CZ) reviewed the articles. Titles and abstracts were reviewed, and SRs were included for full-text review if they assessed at least one PROM specific for arthritis and at least one of the following psychometric properties: validity, reliability, responsiveness, Rasch analysis, factor analysis, cross-cultural validation, interpretability, and floor/ceiling effect. Studies that did not mention any psychometric properties for the outcome measures were excluded from this overview. While acknowledging the nuances among osteoarthritis (OA), RA, and spondylarthritis (SA), our study’s inclusive approach seeks to identify common PROMs.

### Risk of bias assessment

Two authors (CZ and RF) performed the risk of bias (ROB) assessment using the A MeaSurement Tool to Assess systematic Reviews (AMSTAR).^17^ The AMSTAR tool measures the ROB of SRs and comprises 11 items. We considered reviews that scored 8 or above 8 as low risk, between 5 and 7 as moderate risk, and less than 4 as high ROB.^17^

In addition to using the AMSTAR for rating the ROB of each SR, we also extracted and synthesized the type of ROB or quality assessment tool that was used in each primary SR to rate the quality of the primary studies.

### Data extraction

We used a validated data extraction sheet adapted from previous overviews assessing similar outcome assessments.^18-20^ One author performed the data extraction (ECB), which the first author (AD) double-checked. We extracted both the data and the descriptive elements of the study. We extracted data on reported information on the sample size, purpose of the study, patient characteristics, and psychometric properties from the SRs. Original data from the articles included in the SRs were not extracted.

### Statistical analysis

We conducted a qualitative narrative synthesis to report findings on psychometric properties. Owing to the heterogeneity and inconsistency of the included SRs, it was impossible to present a formal statistical analysis. We defined high-quality evidence as where consistent results were mentioned in at least 2 low-to-moderate risks of bias reviews. We described moderate-quality evidence from one or more moderate ROB reviews with consistent reports, whether it was conflicted by high ROB reviews. We defined low-quality evidence from one or more high-risk bias reviews. Finally, we described conflicting evidence when similar quality reviews reported conflicting findings.^18^

## Results

**Figure 1.**
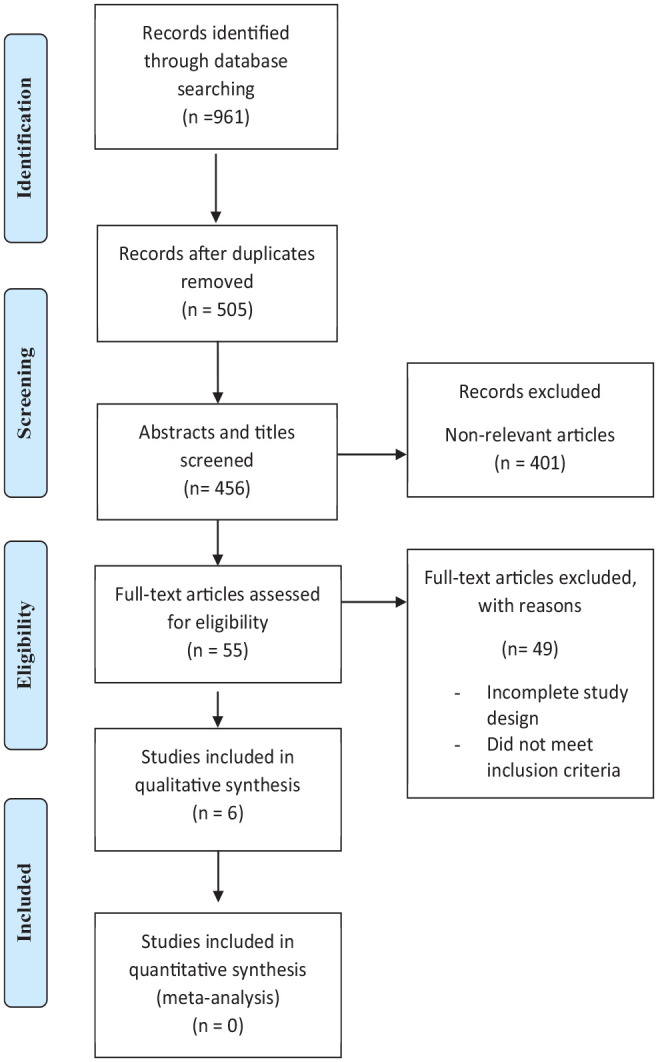
PRISMA flow chart.

Initially, 961 citations were identified by the search of the electronic databases. After removing the duplicates and the prior versions of the updated SRs, 456 titles and abstracts were screened. Fifty-five SRs were deemed eligible for the final review of the full-text articles. Eventually, 6 SRs met the inclusion criteria of this overview and were included in the final synthesis of the evidence (please refer to Figure 1). In all these SRs, a sample of people with upper extremity arthritic disorders was included, and at least one outcome measure specific to arthritic conditions was assessed.

Overall, 36 different arthritis-specific PROMs were reported in the included SRs, out of which 15 could apply to any arthritic condition, 17 were specific for the assessment of RA, 3 were specific to OA, and 3 were specific to ankylosing spondylosis (AS). The characteristics of the included SRs are summarized in Table 1.

**Table 1.** Characteristics of the included systematic reviews.

Authors	Primary relevant studies synthesized	Study population	Arthritis-specific patient-reported outcomes	Results or conclusions^a^	Quality of evidence^a^ (assessment tool)	AMSTAR risk of bias
Alheresh et al^27^	3 (35-37)	RA, OA, AS, Gout	WOJRQ, WPAI-AS, WPS-RA	“None of the instruments had strong quality evidence of criterion validity or responsiveness.”	Highly variable (COSMIN)	Moderate
Eyles et al^21^	1 (38)	OA	SCQOA	“Further well-designed studies investigating measurement properties of existing instruments are required.”	Highly variable (COSMIN)	Moderate
Lee et al^25^	36 (39-73)	RA	AIMS, AIMS-2, AIMS-2 short form, CSHQ-RA, CSHQ-RA short form, Revised CSHQ-RA, QOL-RA, RAID	“The current evidence suggests that the best validated instrument among the RA-specific HRQOL measures is the RAQoL questionnaire in terms of both methodological quality in the process of psychometric evaluation and the quality of the measurement properties. However, there is limited evidence regarding internal consistency and structural validity of the RAQoL. Further efforts are warranted to establish the psychometric quality of this questionnaire.”	Highly variable (COSMIN)	Moderate
Oude Voshaar et al^23^	33 (45,53,57,58,74-102)	RA	AIMS, AIMS short, AIMS shortened, AIMS-2, AIMS-2 short form, CSHQ-RA, Revised CSHQ-RA, CSSRD-FAS, FFbH, HAQ, HAQ-2, modified HAQ, Multidimensional HAQ (10-ADL) and (14-ADL), IRGL, ROAD, SIP-RA	“The disease-specific HAQ and the generic SF-36 can currently be most confidently recommended to measure physical function in RA for most research purposes. The HAQ, however, was frequently associated with considerable ceiling effects while the SF-36 has limited content coverage. Alternative scales that might be better suited for specific research purposes are identified along with future directions for research.”	Highly variable (COSMIN)	High
Swinkels et al^22^	32 (39,42,45,47,76,81-85,103-123)	RA, Seronegative polyarthritis, OA, AS, Polymyositis, FM	AIMS, AIMS-depression, AIMSS-depression, AIMS-anxiety, AIMSS-anxiety, AIMS emotional function, AIMS-pain, AIMS2-pain, AIMSS-pain, AHI, AUSCAN-OHI, ASES, SAJ (swelling), BASDAI, RAPS	“Only a limited number of the identified instruments for the assessment of impairments is both reliable and valid. Allied health care professionals should be cautious in the selection of measurement instruments to assess their patients.”; “For assessment of stiffness, the best instruments are the BASDAI and the VAS-S. For assessment of pain the best instrument is the AIMS.”	Unclear (not reported)	Moderate
Veenhof et al^9^	5 (45,114,124-126)	OA	IRGL, AIMS, AIMS-2, AIMS-2 SF	“Many psychometric qualities were not properly tested for a large number of questionnaires, and none of the questionnaires were rated positive on all aspects of the checklist.”	Ranged from 1 to 4 out of 12 (12-domains checklist)	High

Abbreviations: ADL, Activities of Daily Living; AHI, Arthritis Helplessness Index; AIMS, Arthritis Impact Measurement Scale; AIMSS, AIMS short version; AMSTAR, A MeaSurement Tool to Assess systematic Reviews; AS, ankylosing spondylosis; ASES, Arthritis Self Efficacy Scale; AUSCAN-OHI, Australian/Canadian Osteoarthritis Hand Index; BASDAI, Bath Ankylosing Spondylitis Disease Activity Index; COSMIN, COnsensus-based Standards for the selection of health Measurement INstruments; CSHQ-RA, Cedars-Sinai Health Related Quality of Life for Rheumatoid Arthritis; CSSRD-FAS, Cooperative Systematic Studies for Rheumatic Diseases group Functional Assessment Survey; FFbH, Funktionsfragenbogen, Hannover; FM, Fibromyalgia; HAQ, Health Assessment Questionnaire; HRQoL, health-related quality of life; IRGL, Influence of Rheumatic Disease on General Health and Lifestyle; OA, Osteo Arthritis; QOL-RA, Quality of Life—Rheumatoid Arthritis; RA, Rheumatoid Arthritis; RAID, Rheumatoid Arthritis Impact of Disease; RAPS, Rheumatoid Arthritis Pain Scale; RAQoL, Rheumatoid Arthritis Quality of Life; ROAD, Recent Onset Arthritis Disability Questionnaire; SAJ, Self-Assessment Joint Count; SCQOA, Stages of Change Questionnaire in Osteoarthritis; SF, short form; SIP-RA, Sickness Impact Profile for Rheumatoid Arthritis; WOJRQ, Work Osteoarthritis or Joint Replacement Questionnaire; WPAI-AS, Work Productivity and Activity Impairment Questionnaire for ankylosing spondylitis; WPS-RA, Work Productivity Survey for Rheumatoid Arthritis.

a According to the authors of the reviews.

### Risk-of-bias assessment of SRs

Of the 6 included SRs, 4 had a moderate ROB,^23-26^ and the other 2 had a high ROB.^9,23^ Only 1 of the 6 SRs provided an a priori hypothesis.^21^ Neither of the SRs provided a list of the excluded articles, nor was the likelihood of publication bias assessed. Regarding item 9 on the AMSTAR, 3 SRs used appropriate strategies to combine the findings of their included articles.^21,25,27^ All the SRs provided the characteristics of the included articles, appraised and recorded the quality of the primary studies, and appropriately used this scientific quality in concluding. The AMSTAR quality appraisal table can be found in Supplemental Appendix I.

### Properties of specific PROMs

#### Arthritis Impact Measurement Scale

The psychometric properties of the Arthritis Impact Measurement Scale (AIMS) were assessed by 2 SRs of moderate ROB^22,25^ and 2 SRs of high ROB.^9,23^ Different versions and subscales of AIMS were assessed by these SRs, including AIMS short version, AIMS shortened version, AIMS short form, AIMS2, AIMS2-SF,^24^ AIMS- anxiety subscale, AIMS-pain subscale, and AIMS-depression subscale.^24,26,28-30^

Regarding the reliability of the AIMS, one SR with a moderate ROB reported a pooled test-retest reliability of .86.^22^ Another SR with a high ROB reported internal consistency of α < 0.70 for AIMS physical activity scales and activities of daily living subscales and indeterminate test-retest reliability because of inadequate methodological quality.^23^ The reliability of the AIMS was described as conflicting evidence^22^ in one SR of moderate ROB.

The validity of the AIMS was assessed by 3 SRs.^9,22,23^ Regarding the construct validity of the AIMS, 2 SRs reported consistent findings: Oude Voshaar et al^23^ (high ROB) reported a good construct validity. Similarly, Swinkels et al^22^ (moderate ROB) reported a pooled construct validity of 0.58 (validated against instruments measuring the same impairment) and 0.75 (validated against instruments measuring impairments, disabilities, and participation problems, or general aspects like gender, age). The content validity of the AIMS was described as acceptable in only one SR with a high ROB.^9^

Responsiveness of the AIMS was consistently reported as being unknown in one SR with moderate ROB^25^ and indeterminate in another SR with a high ROB.^9^ In contrast to the findings of these 2 SRs, Oude Voshaar et al (high ROB) reported good responsiveness for the AIMS. The minimal clinically important difference and standard error of the mean were not reported in any SRs.

Regarding the reliability of the AIMS2, 2 SRs reported conflicting findings. While Oude Voshaar et al (high ROB) reported good internal consistency with adequate methodological quality and indeterminate test-retest reliability,^23^ Lee et al (moderate ROB) reported inconclusive reliability due to the conflicting evidence.^25,28,31,32^ Three SRs assessed the validity of the AIMS2. Two of these SRs consistently reported doubtful validity from studies of poor methodological quality.^9,25^ In contrast to these 2 SRs, Oude Voshaar et al^23^ (high ROB) reported good construct validity with studies of adequate methodological quality. Two SRs of moderate and high ROB consistently reported good responsiveness for the AIMS2.^9,25^ The details of the psychometric properties of different variations and subscales of the AIMS can be found in Table 2.

**Table 2.** Summary of the psychometric properties of the AIMS in included systematic reviews.

Instrument	Reliability	Internal consistency	Content validity	Construct validity/correlational	Structural validity	Rasch analysis	Responsiveness
Measurement properties of the AIMS assessed by Lee et al (moderate ROB)^25^							
AIMS	Conflicting evidence	NR	NR	NR	NR	NR	Unknown (only studies of poor methodological quality)
AIMS2	Conflicting evidence	NR	Unknown (only studies of poor methodological quality)	Unknown (only studies of poor methodological quality)	NR	NR	Good
AIMS2-SF	Poor (results from one study with fair methodological quality	NR	NR	Good criterion validity, strongly correlated with MHAQ and SF-36 (*r* = .83, −.73)	NR	NR	Good
Measurement properties of the AIMS assessed by Oude Voshaar et al (high ROB)^23^							
AIMS	Indeterminate because of inadequate methodological quality	Cronbach’s α < .70 for physical activity scales & activities of daily living subscales	NR	Good with adequate methodological quality	NR	NR	Good with adequate methodological quality
AIMS short	Indeterminate because of inadequate methodological quality	Indeterminate, Cronbach’s α < .70	NR	Good with adequate methodological quality	NR	NR	Indeterminate because of inadequate methodological quality
AIMS shortened	Indeterminate because of inadequate methodological quality	Indeterminate internal consistency, Cronbach’s α < 0.70	NR	Poor with adequate methodological quality	NR	NR	No information found for responsiveness
AIMS2	Indeterminate	Good with adequate methodological quality	NR	Good with adequate methodological quality	NR	NR	No information found for responsiveness
AIMS2-SF	Good with adequate methodological quality	Indeterminate	NR	Good with adequate methodological quality	NR	NR	Good with adequate methodological quality
Measurement properties of the AIMS assessed by Veenhof et al (high ROB)^9^							
AIMS	NR	NR	Acceptable	NR	NR	NR	Indeterminate
AIMS2	NR	NR	Doubtful	NR	NR	NR	Indeterminate
AIMS2-SF	NR	NR	Doubtful content validity	NR	NR	NR	NR
Measurement properties of the AIMS assessed by Swinkels et al (moderate ROB)^22^							
AIMS (as a Measurement of impairments in mental functions)	Pooled = 0.86	NR	NR	0.33^a^	NR	NR	NR
AIMS (as a measurement of pain)	Pooled = 0.86	NR	NR	Pooled = 0.58^b^ Pooled = 0.75^a^	NR	NR	NR
AIMS-anxiety	NR	NR	NR	Pooled = 0.43^b^ Pooled = 0.37^a^	NR	NR	NR
AIMS-depri	NR	NR	NR	Pooled = 0.43^b^ 0.28^c^ Pooled = 0.74^a^	NR	NR	NR
AIMS-Emof	NR	NR	NR	Pooled = 0.57^b^ Pooled = 0.19^c^ Pooled = 0.45^a^	NR	NR	NR
AIMSS-anxiety	NR	NR	NR	Pooled = 0.41^b^ Pooled = 0.41^a^	NR	NR	NR
AIMSS-depri	NR	NR	NR	Pooled = 0.47^b^ Pooled = 0.14^c^ Pooled = 0.46^a^	NR	NR	NR
AIMS-pain	Pooled = 0.62	NR	NR	Pooled = 0.40^b^ Pooled = 0.54^d^ Pooled = 0.60^c^	NR	NR	NR
AIMS2-pain	ICC = 0.89	NR	NR	0.49^d^ Pooled = 0.38^c^	NR	NR	NR
AIMSS-pain	Pooled = 0.57	NR	NR	Pooled = 0.41^b^ Pooled = 0.50^c^ Pooled = 0.61^a^	NR	NR	NR

Abbreviations: AIMS, Arthritis Impact Measurement Scale; ICC, Intraclass Correlation Coefficient; MHAQ, Modified Health Assessment Questionnaire; NR, not reported; ROB, risk of bias; SF, short form.

a Validated against instruments measuring impairments, disabilities, and participation problems, or general aspects like, gender, age.

b Validated against instruments measuring same impairment.

c Validated against instruments measuring disabilities.

d Validated against instrument measuring same impairment and other impairments.

#### Cedars-Sinai Health-Related Quality of Life for RA

The psychometric properties of Cedars-Sinai Health-Related Quality of Life for Rheumatoid Arthritis (CSHQ-RA)^33^, revised CSHQ-RA,^34^ and CSHQ-RA short form^35^ were assessed by 2 SRs. One of these SRs had a moderate ROB,^25^ and the other had a high ROB.^23^

Regarding the reliability of the CSHQ-RA, both SRs reported good to excellent internal consistency and test-retest reliability with adequate methodological quality.^23,25^ Regarding its validity, the content validity of the CSHQ-RA was described as excellent by Lee and colleagues (moderate ROB). Still, they did not find enough evidence to report any values for the construct validity.^25^ In contrast to these findings, Oude Voshaar and colleagues (high ROB) reported good construct validity for the CSHQ-RA but did not report on other types of validity.^23^ Finally, according to both SRs, the responsiveness to change of the CSHQ-RA is inconclusive because of the inadequate methodological quality of their included studies.^23,25^

The internal consistency and test-retest reliability of the revised CSHQ-RA were consistently reported as “good with adequate methodological quality” by 2 SRs of moderate and high risks of bias.^23,25^ Regarding the validity of the revised CSHQ-RA, the evidence is contradicting. While Oude Voshaar and colleagues (high ROB) reported a good construct validity with adequate methodological quality,^23^ Lee et al,^25^ in their SR of moderate ROB, reported that there is a lack of evidence to conclude on the validity of the revised CSHQ-RA. Finally, both SRs reported an “indeterminate responsiveness to change” for the revised CSHQ-RA. Specific details of the reliability, validity, and responsiveness of the CSHQ-RA and its revised and short form can be found in Table 3.

**Table 3.** Summary of the psychometric properties of the CSHQ-RA in included systematic reviews.

Instrument	Test-retest reliability	Internal consistency	Content validity	Construct validity/correlational	Structural validity	Rasch analysis	Responsiveness
Measurement properties of the CSHQ-RA assessed by Lee et al (moderate ROB)^25^							
CSHQ-RA	Good	Excellent	Excellent content validity	Not enough evidence in the literature found for construct validity	NR	NR	Indeterminate due to studies of poor methodological quality
CSHQ-RA revised	Good	Good	Limited evidence found supporting good content validity	Not enough evidence in the literature found for construct validity	NR	NR	Indeterminate due to studies of poor methodological quality
CSHQ-RA SF	Good	Good	Good	No data found on construct validity	NR	NR	Indeterminate due to studies of poor methodological quality
Measurement properties of the CSHQ-RA assessed by Oude Voshaar et al (high ROB)^23^							
CSHQ-RA	Good test-retest reliability with adequate methodological quality	Good internal consistency with adequate methodological quality	NR	Good construct validity with adequate methodological quality	NR	NR	Indeterminate responsiveness because of inadequate methodological quality
CSHQ-RA revised	Good test-retest reliability with adequate methodological quality	Good internal consistency with adequate methodological quality	NR	Good construct validity with adequate methodological quality	NR	NR	Indeterminate responsiveness because of inadequate methodological quality

Abbreviations: CSHQ-RA, Cedars-Sinai Health Related Quality of Life for Rheumatoid Arthritis; NR, not report; ROB, risk of bias; SF, short form.

#### Influence of Rheumatic Diseases on General Health and Lifestyle

Two SRs of high ROB assessed the psychometric properties of the English and the Dutch versions of the Influence of Rheumatic Disease on General Health and Lifestyle (IRGL) outcome measure.^9,23^ Both SRs consistently reported inconclusive evidence regarding both versions’ reliability and responsiveness to change.^14,28^ Oude Voshaar and colleagues reported poor construct validity of the Dutch version of the IRGL.^23^

#### Health Assessment Questionnaire

The Health Assessment Questionnaire (HAQ) and its different versions were assessed regarding their validity, reliability, and responsiveness to change by Oude Voshaar and colleagues (high ROB).^23,36-40^ Based on this SR, HAQ has good internal consistency, test-retest reliability, construct validity, and responsiveness to change. Moreover, the modified and the second versions of the HAQ also have good internal consistency, adequate methodological quality, indeterminate test-retest reliability, and good construct validity. The evidence indicates inconclusive psychometric properties for both the 10 & 14 activities of daily living versions of the Multidimensional HAQ.

### Other PROMs for the assessment of arthritis

Table 4 presents the detailed psychometric properties of the outcome measure specific to OA. These outcome measures are the Stages of Change Questionnaire in Osteoarthritis, Work Osteoarthritis or Joint Replacement Questionnaire, and Australian/Canadian Osteoarthritis Hand Index. The evidence on the psychometric properties of PROMs specific to AS was very limited. The Bath Ankylosing Spondylitis Disease Activity Index had a good internal consistency (Cronbach’s α = 0.74) and moderate to good construct validity ranging from 0.57 to 0.70.^22^ Finally, Table 5 summarizes the psychometric properties of PROMs specific to RA.

**Table 4.** Summary of the psychometric properties specific to osteoarthritis in included systematic reviews for osteoarthritis.

Instrument, author, ROB	Test-retest reliability	Internal consistency	Content Validity	Construct validity/correlational	Structural validity	Rasch analysis	Responsiveness
SCQOA, Eyles et al,^21^ moderate ROB	NR	Action α = 0.74 Precontemplation α = 0.70 Contemplator α = 0.77 After removal of 5 items: Action α = 0.79 Precontemplation α = 0.72 Contemplation α = 0.76	NR	Confirmatory Factor Analysis, factor loading & date reduction: removal of items 3, 7, 12, 16, 18 and 20 Principal Components Analysis Repeated Factor Analysis with 15 item scale: 3 factors explained 45% of the variance	NR	NR	NR
WOJRQ, Alheresh et al,^27^ moderate ROB	ICC 0.97 Measurement error, SEM 3.43, smaller than MCID	Cronbach’s α = 0.90	Excellent	Fair construct validity	Fair	NR	Limited evidence available
AUSCAN-OHI, Swinkels et al,^22^ moderate ROB	Pooled ICC 0.84	NR	NR	0.65 (validated against instruments measuring disabilities)	NR	NR	NR

Abbreviations: AUSCAN-OHI, Australian/Canadian Osteoarthritis Hand Index; MCID, Minimal clinically important differences; NR, not reported; ROB, risk of bias; SCQOA, Stages of Change Questionnaire in Osteoarthritis; SEM, Standard Error of Measurement; WOJRQ, Work Osteoarthritis or Joint Replacement Questionnaire.

**Table 5.** Summary of the psychometric properties of WPS-RA, RAID, RAQoL, SIP-RA, ROAD, CSSRD-FAS and FFbH in the included systematic reviews.

	Test-retest reliability	Internal consistency	Content validity	Construct validity/correlational	Structural validity	Rasch analysis	Responsiveness
AHI, Swinkels et al,^22^ moderate ROB	0.53	NR	NR	Pooled = 0.46^a^ Pooled = 0.69^b^ Pooled = of 0.23^c^	NR	NR	NR
AI, Swinkels et al,^22^ moderate ROB	Pooled = 0.80	NR	NR	Pooled = 0.88^a^	NR	NR	NR
ASES, Swinkels et al,^22^ moderate ROB	Pooled = 0.88	NR	NR		NR	NR	NR
CSSRD-FAS, Oude Voshaar et al,^23^ high ROB	ICC < 0.70 for the transfer and mobility scales in stable patients	NR	NR	Good construct validity with adequate methodological quality	NR	NR	Indeterminate (only studies of poor methodological quality)
FFbH, Oude Voshaar et al,^23^ high ROB	No information found on test-retest reliability	Indeterminate internal consistency because of inadequate methodological quality	NR	Good construct validity with adequate methodological quality	NR	NR	Indeterminate (only studies of poor methodological quality)
RAID, Lee et al,^25^ moderate ROB	Good	NR	Excellent	NR	NR	NR	Good
RAQoL, Lee et al,^25^ moderate ROB	Excellent	Limited evidence supporting poor internal consistency	Excellent	Limited evidence supporting poor construct validity Indeterminate criterion validity (only studies of poor methodological quality)		NR	Good
RAPS, Swinkels et al,^22^ moderate ROB	0.92	NR	NR	0.52, 0.68^a^	NR	NR	NR
ROAD, Oude Voshaar et al,^23^ high ROB	Good	Good	NR	Subscales inadequately related to HAQ (*r* = 0.17, −0.32) and SF-36 (*r* = 0.18-0.32) Poor construct validity		NR	Good responsiveness with adequate methodological quality
SAJ (sensory), Swinkels et al,^22^ moderate ROB	Pooled = 0.82	NR	NR	0.55^a^	NR	NR	NR
SIP-RA, Oude Voshaar et al,^23^ high ROB	Indeterminate (only studies of poor methodological quality)	NR	NR	NR	NR	NR	Indeterminate (only studies of poor methodological quality)
Stest, Swinkels et al,^22^ moderate ROB	Pooled = 0.67	NR	NR		NR	NR	NR

Abbreviations: AHI, Arthritis Helplessness Index; AI, Articular Index; ASES, Arthritis Self Efficacy Scale; CSSRD-FAS, Cooperative Systematic Studies for Rheumatic Diseases group Functional Assessment Survey; FFbH, Funktionsfragenbogen, Hannover; HAQ, Health Assessment Questionnaire; NR, not reported; RAID, Rheumatoid Arthritis Impact of Disease; RAPS, Rheumatoid Arthritis Pain Scale; RAQoL, Rheumatoid Arthritis Quality of Life; ROAD, Recent Onset Arthritis Disability Questionnaire; ROB, risk of bias; SAJ, Self-Assessment Joint count; SF, short form; SIP-RA, Sickness Impact Profile for Rheumatoid Arthritis; Stest, Stiffness test; WPS-RA, Work Productivity Survey for Rheumatoid Arthritis.

a Validated against instruments measuring impairments, disabilities, and participation problems, or general aspects like, gender, age.

b Validated against instruments measuring same impairment.

c Validated against instruments measuring disabilities.

## Discussion

This overview of SRs systematically synthesized the current state of knowledge for 36 arthritis-specific PROMs. This overview identified publication bias in the SRs and that the evidence was inconclusive or missing for various measurement properties for most measures. Low- to moderate-quality evidence was found of good construct and criterion validity, acceptable content validity, and good responsiveness of the AIMS. We found low to moderate-quality evidence of good internal consistency, test-retest reliability, and construct validity of the CSHQ-RA. Disease-specific measures for arthritis were the focus of this overview since clinicians must choose between regional and disease-specific measures when deciding which PROMs are best for their patients with arthritis. Given the strong evidence for regional-specific measures like the DASH and PRWE and the lack of head-to-head comparisons, this overview could not address whether a disease-specific measure is preferable to a generic measure for patients with different types of arthritis.

Overlap in the studies included in our SRs was minimal. Therefore, we trust that this level of overlap does not significantly affect the presentation or interpretation of our results. The included SRs represented several arthritic conditions, the most common being RA and OA.^9^,^21-23,25,27^ Given that we restricted our overview to studies that only included arthritis-specific PROMs, the current state of the literature demonstrates a lack of high-quality evidence for these conditions. Even though the psychometric properties of PROMs for RA and OA populations were assessed in several SRs, given the highly variable quality of the evidence, it was impossible to generate a quick clinical decision-making recommendation.

The workgroup Outcome Measures in Rheumatoid Arthritis Clinical Trials emphasizes the methodological quality of the PROMs.^41^ Our synthesis detected a moderate to high ROB in reporting the included studies, which were assessed according to the AMSTAR checklist. Even though only 2 of the included SRs demonstrated a high ROB—a number that cannot lead to deductions or conclusions about the literature in general—an interesting observation is that these studies were published before 2011 when tools such as AMSTAR were not widely used.^17^ The main sources of bias found by our overview were summarized in 3 important categories: (1) the included SRs failed to provide a complete list of included and excluded studies, (2) they did not assess the likelihood of publication bias, and (3) they did not provide “a priori” study design. All authors in the included reviews provided the characteristics of the included studies, assessed and reported the scientific quality of the information they summarized, and used the quality-related information to appraise the existing literature and form conclusions critically. Future researchers conducting primary measurement research studies should adhere to the best quality research design and reporting guidelines to ensure the contact and reporting of the measurement property findings provide more definitive and comprehensive conclusions about the measurement properties of different PROMs as COSMIN,^42^ researchers not only meet adequate quality standards for evaluating and reporting their findings, they will also create tools based on advanced transparency and knowledge synthesis.

Our analysis’s absence of recent SRs highlights potential gaps in current research on PROMs for upper extremity arthritis. The limited inclusion of SRs since 2017 might stem from a slower research pace in this domain, involving methodologies requiring alignment or a paucity of primary studies addressing PROMs. Acknowledging that this gap impacts the comprehensiveness of our findings, this overview offers valuable insights into the existing SRs, emphasizing the need for up-to-date research to guide clinical practice and enhance patient-centered care in this field.

Reliability is an essential and fundamental measurement property that tells clinicians the extent to which they can expect stable scores when the patient status remains unchanged.^9^ As for the reliability of the assessed PROMs, evidence indicated good to excellent test-retest reliability and internal consistency of the CSHQ-RA, RAQol, and AUSCAN-OHI (Pooled ICC = 0.84) and good test-retest reliability and internal consistency of the HAQ, ROAD, and BASDAI (ICC = 0.74), inconclusive test-retest reliability of the AIMS, AIMS2, AIMS-2-SF, and IRGL. The remaining PROMs had either no evidence or poor reliability and internal consistency. Internal consistency was reported for 20 of the 36 PROMs and that is seemingly a high number at the surface; however, the SRs either did not report the Cronbach’s alpha value, or the Cronbach’s alpha value was less than 0.70 for 16 of the PROMs, and this indicates poor internal consistency.

The validity, defined as the extent to which an instrument measures the construct it is supposed to measure,^9^ is another critical psychometric property to consider in clinical decision-making. A major issue in our overview was finding substantial gaps in reporting the validity of the arthritis-specific PROMs. Construct validity was the most frequently reported type but often consisted of correlations between different PROMs without prespecified hypotheses. When arthritis-specific PROMs were correlated against other more generic PROMs, such as SF-36, the SRs found insufficient evidence in the literature for construct validity or reported weak to moderate correlations. Only one SR (Swinkels et al) categorized the construct validity of the AIMS and its variations based on the type of instrument they were validated against, where the pooled correlation for all of them indicated weak to moderate construct validity, except for AIMS depression (pooled correlation = 0.74). Content, face, criterion, and other types of validity that might have provided more rigorous indicators were rarely reported by the SRs. Content validity through qualitative or quantitative content validity indices has not been reported, which is one of the most important types of validity when interpreting the comprehensibility and comprehensiveness of any PROM,^43^ and we recommend that future research should focus on this gap. Factorial validity through Rasch analysis was only reported for HAQ-II, and even in that case, there was no evidence for assessment of the dimensionality of the scales beyond the reporting of item-level fit statistics.

The responsiveness of a PROM, as defined by Kirshner and Guyatt, is a measure’s ability to detect a clinically important difference.^44^ It is one of the most important measurements for clinicians because they often want to determine whether patients recover after treatment.^44^ In this overview, several SRs reported effect sizes, MCID, and SRM values to examine if the amount of change that occurred is clinically meaningful/important. Responsiveness was assessed for only 7 of the 36 PROMs, and where it was studied, it was often through low-quality evidence. For this reason, we could not make conclusions about most of the measures of responsiveness. Considering the clinical value of the responsiveness of a PROM, failures to evaluate and report the property of responsiveness result in a gap in knowledge and hinder the effective transition of the PROMs into clinical practice.

### Limitations

The current overview has limitations that one must consider before interpreting our findings. First, we acknowledge that the AMSTAR ROB tool may not critically appraise and capture all the relevant and important aspects of SRs; hence, we did not evaluate the quality of each primarily included study in the SRs. Instead, we extracted the ROB or quality assessment reported in each primary SR. Furthermore, the methodology of overviews requires that the measurement properties and the conclusions determined within the original SRs be reported, and it is possible that the original SR incorrectly interpreted the primary findings of some studies. In this overview, we noted a minimal overlap in the primary included studies within the SRs, which means a minimal overlap of the measurement properties of the PROMs. Even though these SRs had different purposes, populations, and eligibility criteria, this overlap led to conclusions that were almost consistent within SRs. Third, despite conducting an electronic search of 6 databases, it is possible that we have missed other SRs on the topic. Finally, perhaps the most important limitation for clinicians is that the SRs did not address head-to-head comparison of the evidence on arthritis-specific measures with upper extremity, PROMs commonly used in practice such as DASH and PRWE. Therefore, we cannot conclude whether an arthritis-specific measure PROM has advantages over these commonly used measures.

### Implications for research and practice

Our overview identified several gaps in the literature, leading to considerable key areas for improvement in future research. First, by applying the AMSTAR ROB tool, we identified several issues such as (1) including a list of the included and excluded studies, (2) providing a priori design, (3) performing a comprehensive literature search; and (4) assessing the likelihood of publication bias. Furthermore, future primary clinical measurement research studies should include more head-to-head comparisons of different PROMs in the same clinical context and the same population to determine their clinical performance. It would be particularly important to compare upper extremity and arthritis-specific measures in arthritis populations. Content validity, structural validity, and responsiveness have all been inadequately addressed in these measures and require high-quality studies.

## Conclusion

Overall, this overview found a moderate to high ROB in the included SRs on the upper extremity PROMs designed specifically for patients with arthritis. Most of the PROMs lacked vigorous psychometric assessments and head-to-head comparisons with commonly used upper extremity measures were largely under-reported by the included SRs.

## Supplemental Material

sj-docx-1-amd-10.1177_11795441231213887 – Supplemental material for Patient-Reported Outcome Measures for Patients with Upper Extremity Arthritis: Overview of Systematic Reviews Click here for additional data file. Supplemental material, sj-docx-1-amd-10.1177_11795441231213887 for Patient-Reported Outcome Measures for Patients with Upper Extremity Arthritis: Overview of Systematic Reviews by Armaghan Dabbagh, Christina Ziebart, Rochelle Furtado, Eleni C Boutsikari and Joy C MacDermid in Clinical Medicine Insights: Arthritis and Musculoskeletal Disorders

sj-docx-3-amd-10.1177_11795441231213887 – Supplemental material for Patient-Reported Outcome Measures for Patients with Upper Extremity Arthritis: Overview of Systematic Reviews Click here for additional data file. Supplemental material, sj-docx-3-amd-10.1177_11795441231213887 for Patient-Reported Outcome Measures for Patients with Upper Extremity Arthritis: Overview of Systematic Reviews by Armaghan Dabbagh, Christina Ziebart, Rochelle Furtado, Eleni C Boutsikari and Joy C MacDermid in Clinical Medicine Insights: Arthritis and Musculoskeletal Disorders

sj-xlsx-2-amd-10.1177_11795441231213887 – Supplemental material for Patient-Reported Outcome Measures for Patients with Upper Extremity Arthritis: Overview of Systematic Reviews Click here for additional data file. Supplemental material, sj-xlsx-2-amd-10.1177_11795441231213887 for Patient-Reported Outcome Measures for Patients with Upper Extremity Arthritis: Overview of Systematic Reviews by Armaghan Dabbagh, Christina Ziebart, Rochelle Furtado, Eleni C Boutsikari and Joy C MacDermid in Clinical Medicine Insights: Arthritis and Musculoskeletal Disorders

## Declarations

**Ethics Approval and Consent to Participate:** Not applicable.

**Consent for Publication:** Not applicable

**Availability of Data and Materials:** All study data are presented in the study.
