# Supplementary material for: Patient-Reported Outcome Measures for Patients with Upper Extremity Arthritis: Overview of Systematic Reviews
Source: Clin Med Insights Arthritis Musculoskelet Disord. 2023 Dec 6;16:11795441231213887. doi: 10.1177/11795441231213887 (PMC10702415; doi:10.1177/11795441231213887)
Supplement: sj-docx-1-amd-10.1177_11795441231213887 – Supplemental material for Patient-Reported Outcome Measures for Patients with Upper Extremity Arthritis: Overview of Systematic Reviews [file sj-docx-1-amd-10.1177_11795441231213887.docx]

Arthritis Overview AMSTAR

| Systematic Reviews | 1 | 2 | 3 | 4 | 5 | 6 | 7 | 8 | 9 | 10 | 11 | Risk of Bias* | Quality of evidence^ (assessment tool) |
| --- | --- | --- | --- | --- | --- | --- | --- | --- | --- | --- | --- | --- | --- |
| Alheresh, 2016 | N | Y | N | N | N | Y | Y | Y | Y | N | N | Moderate | Highly variable  (COSMIN) |
| Eyles, 2017 | Y | Y | N | N | N | Y | Y | Y | Y | N | Y | Moderate | Highly variable  (COSMIN) |
| Lee, 2014 | N | Y | N | N | N | Y | Y | Y | Y | N | Y | Moderate | Highly variable  (COSMIN) |
| Oude Voshaar, 2011 | N | Y | N | N | N | Y | Y | Y | N | N | N | High | Highly variable  (COSMIN) |
| Swinkles, 2005 | N | Y | Y | Y | N | Y | Y | Y | N | N | N | Moderate | Unclear (not reported) |
| Veenhof, 2006 | N | Y | N | N | N | Y | Y | Y | N | N | N | High | Ranged from 1 to 4 out of 12 (12-domains checklist) |

***List of Abbreviations:*** *AMSTAR, A MeaSurement Tool to Assess systematic Reviews; N, no; Y, yes.* COSMIN, Consensus-Based Standards for the Selection of Health Measurement Instruments.

**Scores of 8 or higher were considered as low risk of bias, scores of 5 through 7 as moderate risk of bias, and scores of 4 or less as high risk of bias.*

^ *According to the authors of the reviews*

*1, Was an a priori design provided? 2, Was there duplicate study selection and data extraction? 3, Was a comprehensive literature search performed? 4, Was the status of publication (i.e., gray literature) used as an inclusion criterion? 5, Was a list of studies (included and excluded) provided? 6, Were the characteris­tics of the included studies provided? 7, Was the scientific quality of the included studies assessed and documented? 8, Was the scientific quality of the included studies used appropriately in formulating conclusions? 9, Were the methods used to combine the findings of studies appropriate? 10, Was the likelihood of publication bias assessed? 11, Was the conflict of interest included?*
